# Structural investigations of benzoyl fluoride and the benzoacyl cation of low-melting com­pounds and reactive inter­mediates

**DOI:** 10.1107/S2053229625000476

**Published:** 2025-01-24

**Authors:** Valentin Bockmair, Martin Regnat, Huu Khanh Trinh Tran, Andreas J. Kornath

**Affiliations:** a Department Chemie, Ludwig-Maximilians Universität, Butenandtstrasse 5-13 (Haus D), D-81377 München, Germany; University of Notre Dame, USA

**Keywords:** crystal structure, benzo­acyl­ium, benzoyl fluoride, acyl cation, acyl fluoride

## Abstract

Acyl fluorides and acyl cations represent typical reactive inter­mediates in organic reactions, such as Friedel–Crafts acyl­ation. However, the com­paratively stable phenyl-substituted com­pounds have not been fully characterized yet, offering a promising backbone. Attempts to isolate the benzo­acyl­ium cation have only been carried out starting from the acyl chloride with weaker chloride-based Lewis acids. Therefore, only adducts of 1,4-stabilized acyl cations could be obtained. Due to the low melting point of benzoyl fluoride, together with its volitality and sensitivity toward hydrolysis, the structures of the acyl fluoride and its acyl­ium cation have not been determined. Herein, we report the first crystal structure of benzoyl fluoride, C_7_H_5_FO or PhCOF (monoclinic *P*2_1_/*n*, *Z* = 8) and the benzo­acyl­ium undeca­fluoro­diarsenate, C_7_H_5_O^+^·As_2_F_11_^−^ or [PhCO]^+^[As_2_F_11_]^−^ (monoclinic *P*2_1_/*n*, *Z* = 4). The com­pounds were characterized by low-tem­per­a­ture vibrational spectroscopy and single-crystal X-ray analysis, and are discussed together with quantum chemical calculations. In addition, their specific π-inter­actions were elucidated.

## Abstract 2

Acyl cations exist as short-living species in organic reactions starting from the corresponding acyl chloride or acyl fluoride. Benzoyl fluoride and the benzo­acyl­ium cation are highly reactive and sensitive com­pounds. The first structure determination of the low-melting fluoride and its corresponding acyl­ium cation uncovers their crystal structures, built up by various C⋯F contacts, as well as different kind of π-inter­actions.

## Introduction

Benzoyl fluoride, the acyl fluoride of benzoic acid, was first described in the mid-19th century (Borodine, 1863 ▸). Although vibrational spectroscopy (Seewann-Albert & Kahovec, 1948 ▸; Green & Harrison, 1977 ▸; Kniseley *et al.*, 1962 ▸; Kakar, 1972 ▸) and theroetical calculations concerning the inter­nal rotational barrier (Yadav *et al.*, 1987 ▸) were reported in the literature decades ago, the com­pound has not been structurally characterized, presumably due to its low melting point of 244.5 K (Jander & Schwiegk, 1961 ▸) and high sensitivity towards hydrolysis. The appropriate material properties of benzoyl fluoride make it essential as a construction material and depolymerization agent for silicones.

In contrast to the related acyl halides, benzoyl fluoride posesses low electrical conductivity, estimated to be due to self-dissociation (Scheme 1) as reported by Jander & Schwiegk (1961 ▸), which makes the com­pound a potent ionic liquid. The source of the conductivity was assumed to be the formation of the benzo­acyl­ium cation. The addition of a strong Lewis acid (*L*) to benzoyl fluoride resulted in a signifcant increase of the conductivity, which was referred to as the benzoyl cation, as well as *L*F after fluoride abstraction.

The trapping of these reactive aromatic inter­mediates of Friedel–Crafts acyl­ation was further investigated in modern research to isolate the benzoyl chloride anti­mony penta­chloride adduct, as well as the toluenacyl­ium cation (Davlieva *et al.*, 2005 ▸). Nevertheless, despite much effort, the crystal structure of benzoyl fluoride and the respective acyl­ium ion could not be determined. Similar attempts were made to characterize the 1,4-di­acyl­ium cation of benzene (Olah & Comisarow, 1966 ▸). This raises the question whether a stabilizing effect of the *para* substitutent is needed for the ab­straction of the halogen ion or only for acyl chlorides, as reported previously (Davlieva *et al.*, 2005 ▸).
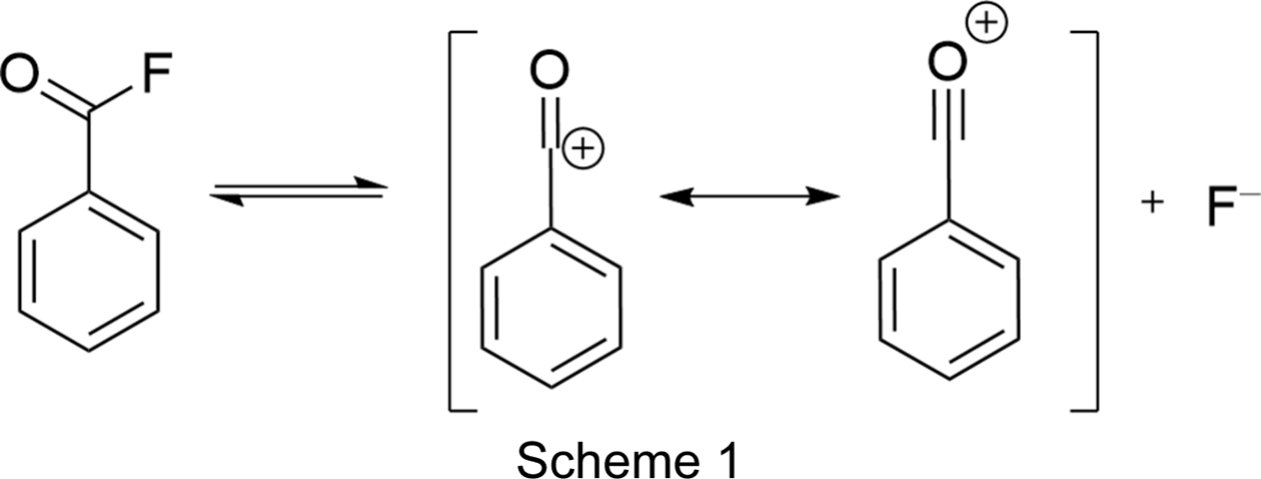


Although there are many ways to synthesize benzoyl fluoride, a catalyst-free path was chosen. The synthesis path from benzoic acid with sulfur tetra­fluoride was preferred, yielding benzoyl fluoride in high purity, only containing volatile by-products (Scheme 2). Arsenic penta­fluoride was used for fluoride trapping due to its high fluoride ion affinity.

Besides the benzoic acid derivatives, investigations of the fluorinate and acyl­ate terephthalic acid and isophthalic acid were performed to com­pare the stability and influence of the respective moieties on the aromatic system.

## Experimental

**Caution!** Note that any contact with the described com­pounds should be avoided. Hydrolysis of AsF_5_, SF_4_, SOF_2_ and the synthesized salts forms HF which burns the skin and causes irreparable damage. Safety precautions should be taken while handling these com­pounds.
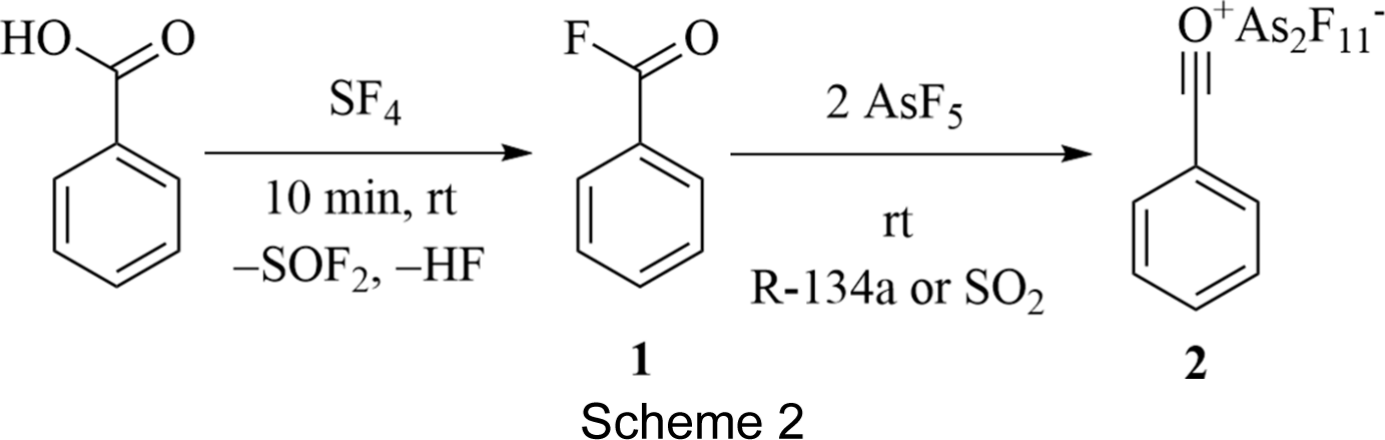


All reactions were carried out by employing standard Schlenk techniques on a stainless steel vacuum line. The syn­theses of the salts were performed using FEP (fluorinated ethyl­ene–propyl­ene copolymer)/PFA (perfluoro­alk­oxy­al­kane) reactors with stainless steel valves.

### Synthesis and crystallization

Benzoic acid (65 mg, 0.532 mmol, 1 equiv.) was added to an FEP reactor in a nitro­gen countercurrent flow. Sulfur tetra­fluoride (116 mg, 1.07 mmol, 2 equiv.) was then condensed in a static vacuum in the reactor and frozen with liquid nitro­gen. The reaction mixture was warmed to room tem­per­a­ture and homogenized until liquified. The generated thionyl fluoride and hy­dro­gen fluoride were removed in a dynamic vacuum at 195 K. Benzoyl fluoride (**1**) was obtained as a colourless solid in qu­anti­tative yield.

For the crystallization of benzoyl fluoride (**1**), the crude product was recrystallized at 195 K under a cooled nitro­gen stream to remove the last traces of thionyl fluoride and to solidify the saturated solution.

Arsenic penta­fluoride (904 mg, 5.32 mmol, 10 equiv.) was condensed in a static vacuum in the FEP reactor containing synthesized benzoyl fluoride (**1**) and then frozen with liquid nitro­gen. Sulfur dioxide (2 ml) was condensed in the reactor and frozen in a static vacuum. The reaction mixture was warmed to room tem­per­a­ture and homogenized until the solution was clear. After the removal of excess arsenic pen­ta­­fluoride and solvent, benzo­acyl­ium undeca­fluoro­diarsenate (**2**) was obtained as a colourless solid in qu­anti­tative yield.

## Analysis

The products PhCOF (**1**) and [PhCO][As_2_F_11_] (**2**) were characterized by single-crystal X-ray diffraction and low-tem­per­a­ture vibrational spectroscopy. In addition, quantum chemical calculations were perfomed with *GAUSSIAN* (Frisch *et al.*, 2016 ▸) to com­pare the observed frequencies and bond lengths, as well as displaying the mapped electrostatic potential using *GaussView* (Dennington *et al.*, 2016 ▸).

Single crystals of **1** and **2** suitable for single-crystal diffraction analysis were selected under a stereo microscope in a cooled nitro­gen stream. Single crystals were prepared on a stainless steel polyamide micromount and the data collections were performed at 112 and 114 K, respectively, on an Xcalibur diffractometer system (Rigaku Oxford Diffraction). Details of the data collection and treatment, as well as structure solution and refinement, are available in the CIF in the supporting information.

**Figure 1:**
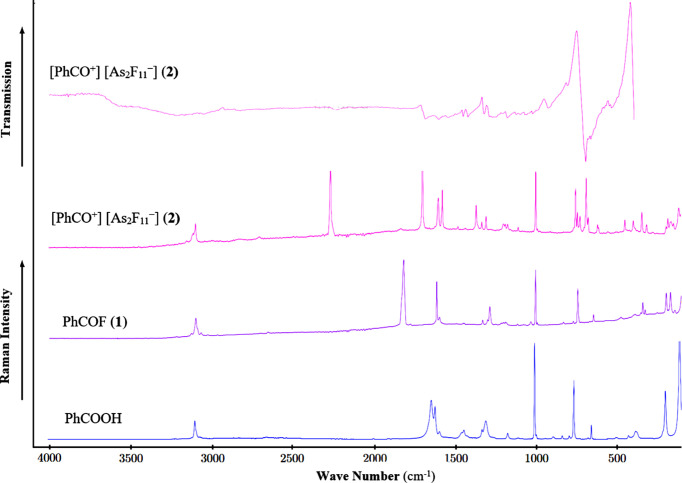
IR and Raman spectra of PhCOOH, PhCOF (**1**) and benzo­acyl­ium undeca­fluoro­diarsenate (**2**).

Low-tem­per­a­ture vibrational spectroscopy measurements were performed to screen the conversion. IR spectroscopic investigations were carried out with a Bruker Vertex-80V FT–IR spectrometer using a cooled cell with a single-crystal CsBr plate on which small amounts of the samples were placed (Bayersdorfer *et al.*, 1972 ▸). For Raman measurements, a Bruker MultiRam FT–Raman spectrometer with Nd:YAG laser excitation (λ = 1064 nm) was used. The measurement was performed after transferring the sample to a cooled (77 K) glass cell under a nitro­gen atmosphere and subsequent evacuation of the glass cell. The low-tem­per­a­ture IR spectra are depicted in Fig. 1 ▸.

### Crystal structure refinement

Basic crystallographic data and details of the data collection and structure refinement are summarized in Table 1 ▸ (Sheldrick, 2015*b* ▸). For benzoyl fluoride (**1**), an alert for the Hir­sh­feld test was reported by *PLATON* (Spek, 2020 ▸). Therefore, a disordered O/F (*A*; 50:50 occupancy ratio) model was applied, improving the model com­pared with an ordered sys­tem in the course of structure refminement. The positions of the H atoms in the structure were localized in the difference Fourier map and refined without any restrictions. All atoms occupy the general position 4*e*.

For the refinement of the H-atom positions in the structure of [PhCO][As_2_F_11_] (**2**), the positions were localized from a difference Fourier map and refined without any restraints, with the exception of atom H3, which was idealized for an aromatic C—H distance and angles. All atoms occupy the general position 4*e*.

### Crystal structure

**Figure 2:**
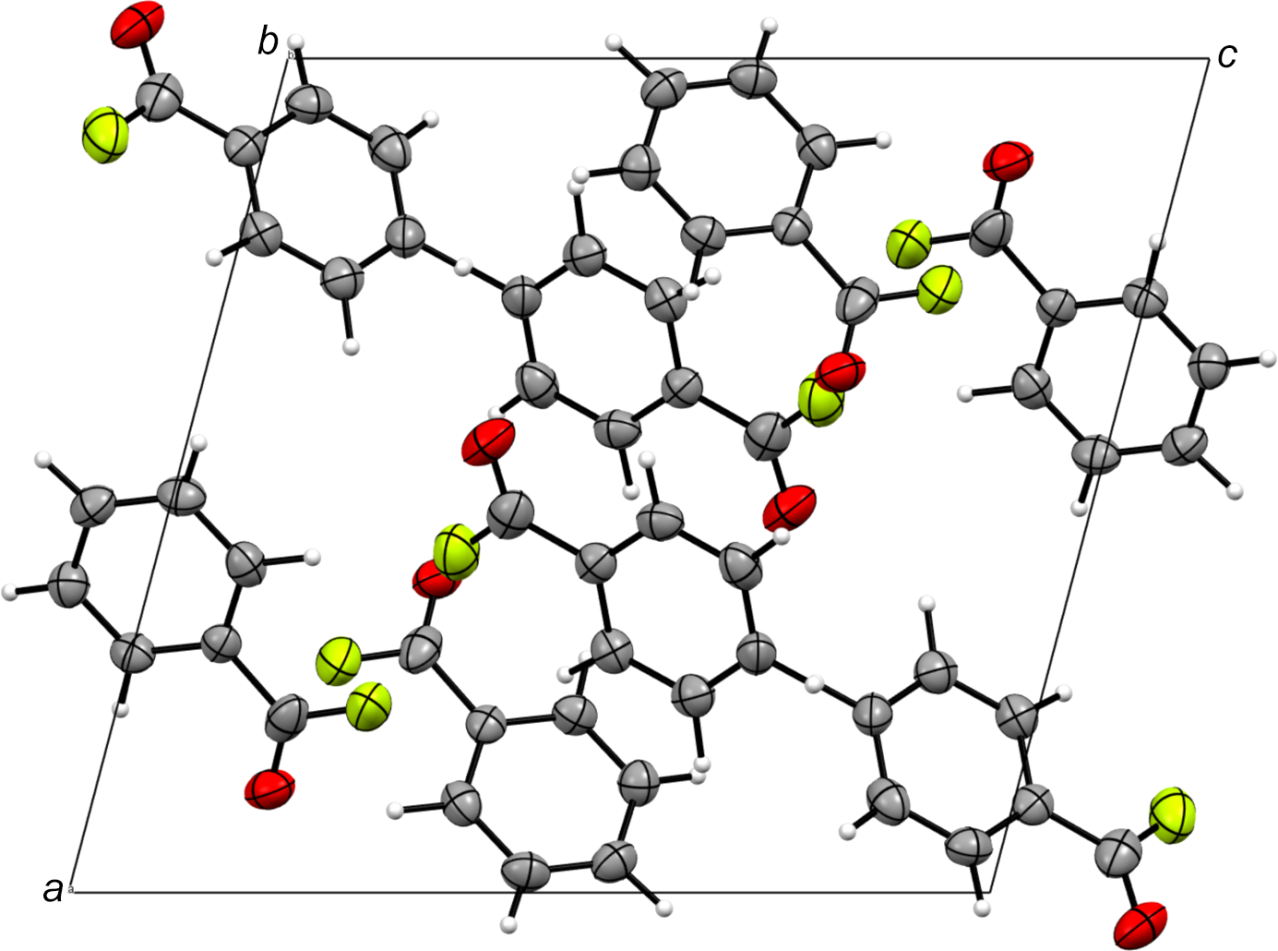
Crystal structure of benzoyl fluoride (**1**), viewed along the *b* axis. Displacement ellipsoids are drawn at the 50% probability level.

**Figure 3:**
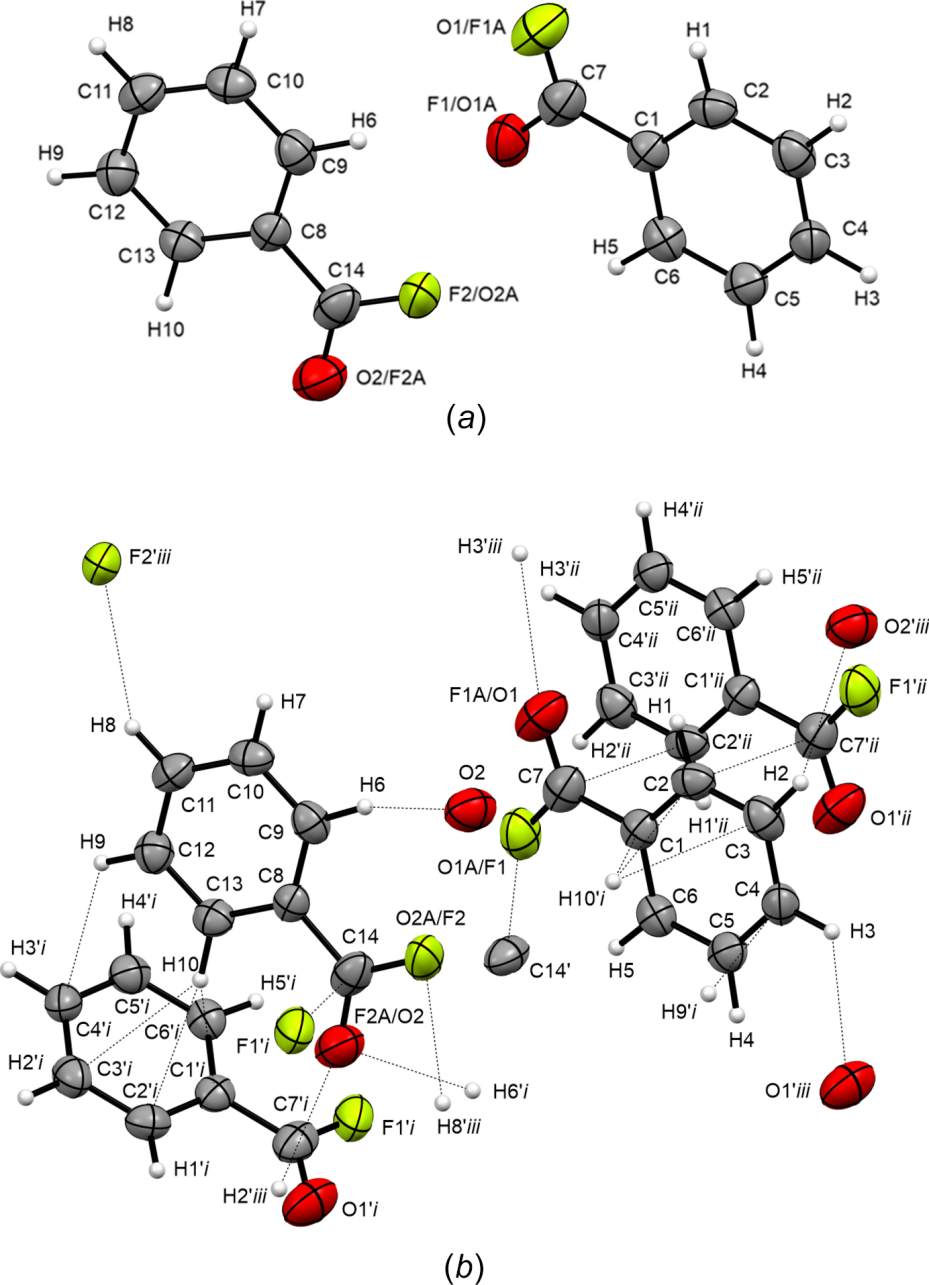
The asymmetric unit of (*a*) benzoyl fluoride (**1**) and (*b*) its short contacts with neighbouring mol­ecules (′). Displacement ellipsoids are drawn at the 50% probability level. [Symmetry codes: (i) −*x* + 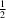, *y* + 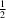, −*z* + 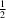; (ii) −*x*, −*y*, −*z*; (iii) *x* + 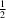, −*y* + 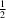, *z* + 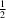.]

Benzoyl fluoride (**1**) crystallizes in the monoclinic space group *P*2_1_/*n*, with eight formula units per unit cell (Fig. 2 ▸). The asymmetric unit of **1** [Fig. 3 ▸(*a*)] is built up of two crystallographically independent mol­ecules, with different chemical enviroments [Fig. 3 ▸(*b*)]. The two rings are formed by atoms C1–C6 and C8–C13. Benzoyl fluoride shows similar C—C bond lengths to benzoic acid and benzoyl chloride, considering the aromatic ring, as reported in Table 2 ▸. When the electron-withdrawing effect of the substituent is increased by converting the carb­oxy­lic acid group to acyl halogenide, the C_Ph_—C bond is significantly shortened. The COF moiety has C=O bond lengths of 1.222 (4) and 1.224 (4) Å, whereas the C—F bond length is com­paratively short with respect to already known acyl fluorides, with values of 1.296 (5) and 1.312 (4) Å (Durig *et al.*, 1998 ▸; van Eijck *et al.*, 1977 ▸; Bayer *et al.*, 2022*a* ▸,*b* ▸). This phenomenon can be rationalized by strong hyperconjugative effects of the arene ring on atom C7, but as the two rings in the asymmetric unit form different weak contacts, small deviations in the C—F bond lengths can be detected. The angles within the benzylic ring are within the 3σ rule [119.2 (3)–120.8 (3)°] and can therefore be regarded as idealized 120° angles in both parts of the asymmetric unit.

In the crystal structure, benzoyl fluoride is mainly stabilized either by C⋯*A* (*A* = O or F) inter­actions or aromatic inter­actions, as listed in Table 3 ▸. These contacts are 3.092 (4) (*A*1⋯C14), 3.295 (3) (*A*1⋯H3—C4), 3.398 (5) (*A*2⋯H2—C3), 3.461 (3) (*A*2⋯C11) and 3.520 (4) Å (O2⋯H6—C9). In addition, strong inter­actions of the π-systems were detected by parallel-displaced stacking at a distance of 3.328 Å (C2⋯C7^i^) along the inversion centre at [0,0,0], and a T-shaped medium inter­action (C13—H10⋯π; π–σ attraction) was detected at a distance of 3.476 Å (C13⋯*Cg*1; *Cg*1 is the centroid of the ring), as the C—H bond is tilted 30.14° with respect to the ring normal (Janiac, 2000 ▸).

**Figure 4:**
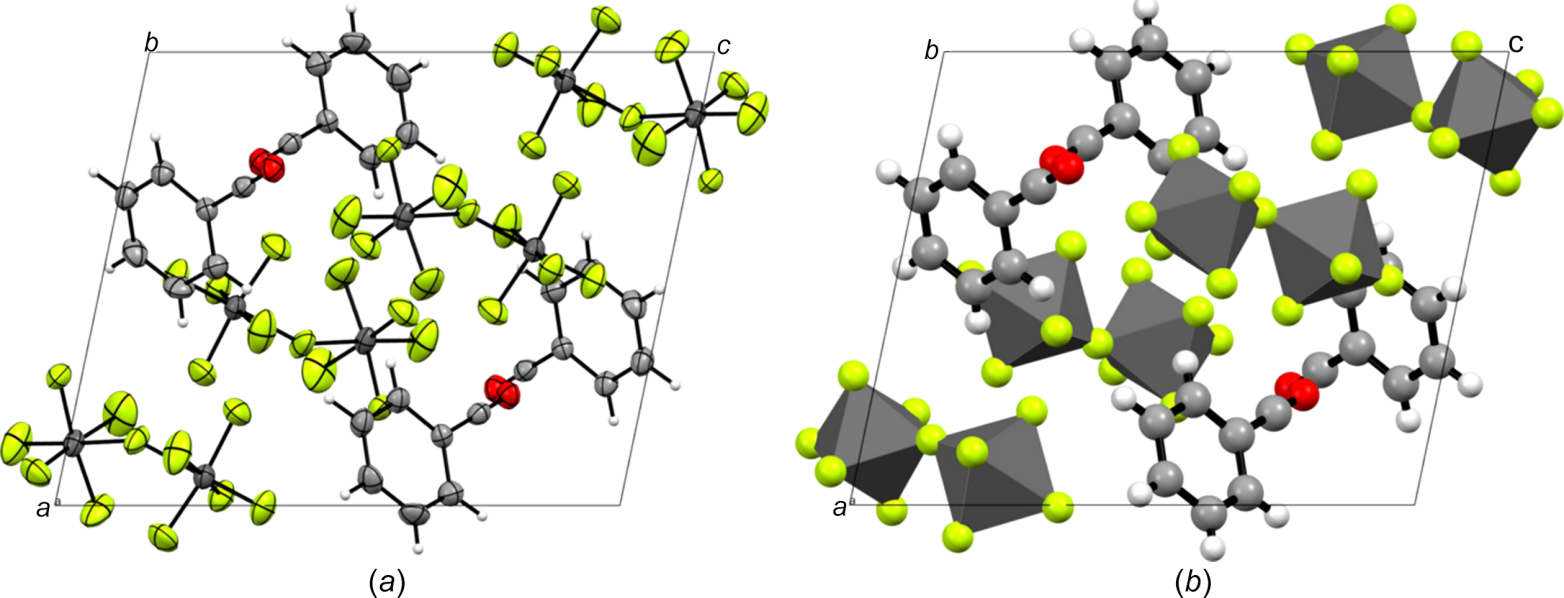
The crystal structure of benzo­acyl­ium undeca­fluoro­diarsenate (**2**), viewed along the *b* axis, (*a*) with displacement ellipsoids and (*b*) in a polyhedral illustration. Displacement ellipsoids are drawn at the 50% probability level.

**Figure 5:**
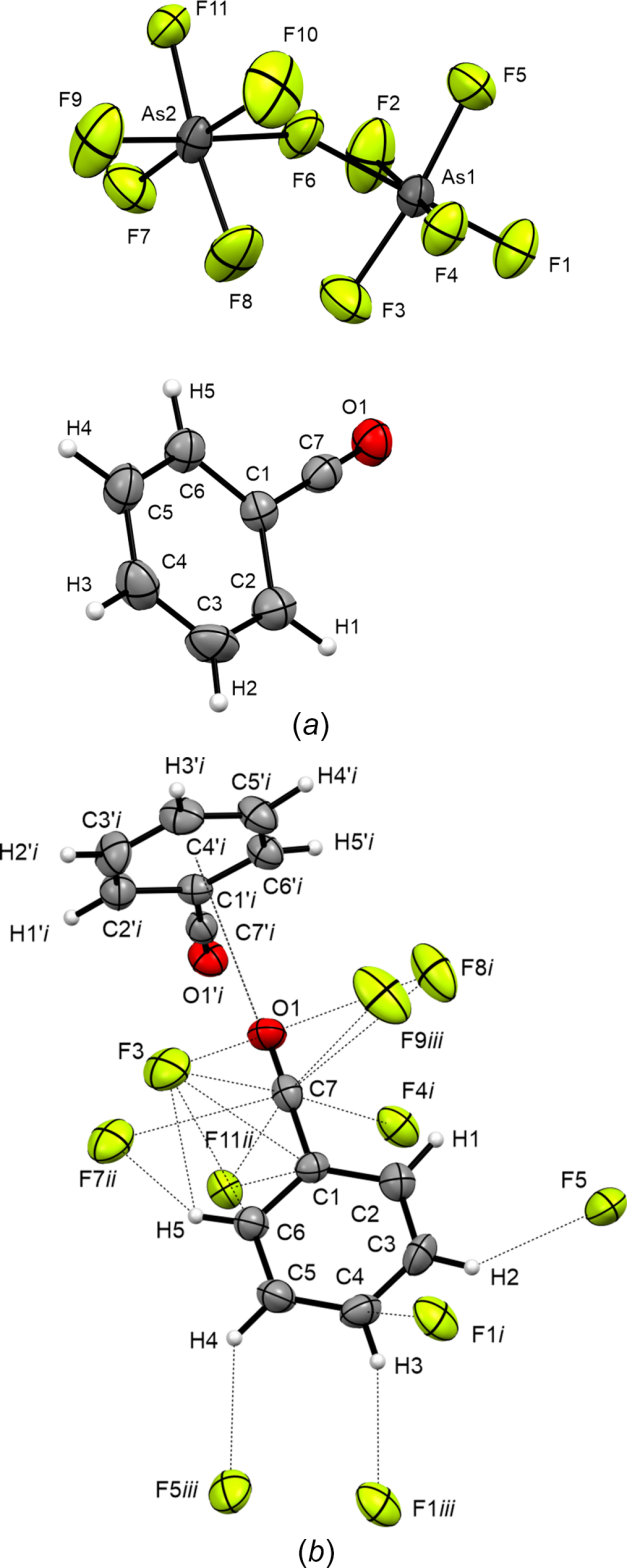
(*a*) The asymmetric unit of [PhCO][As_2_F_11_] (**2**) and (*b*) short contacts of the benzo­acyl­ium cation. Displacement ellipsoids are drawn at the 50% probability level. [Symmetry codes: (i) −*x* + 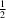, *y* + 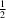, −*z* + 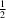; (ii) −*x*, −*y*, −*z*; (iii) *x* + 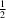, −*y* + 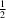, *z* + 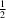.]

Benzo­acyl­ium undeca­fluoro­diarsenate (**2**) crystallizes in the monoclinic space group *P*2_1_/*n*, with four formula units per unit cell (Fig. 4 ▸). The asymmetric unit [Fig. 5 ▸(*a*)] is built up of one PhCO^+^ cation and one As_2_F11^−^ anion. The C≡O bond length is in accordance with known bond lengths of acyl­ium com­pounds, such as the CH_3_CO^+^ cation (Table 4 ▸; Boer, 1966 ▸), whereas the C—C bond is significantly elongated. Regarding the C_Ph_—C bond length of 1.472 (4) Å in **2**, this bond is significantly shortened to 1.403 (5) Å in **1** by the stabilizing mesomeric effects of the π-system. The C—C bond lengths within the arene ring are similar to those of **1**. The angles in the arene ring are close to the idealized angle (120°) and are in the range 117.4 (4)–122.8 (3)°. The bond lengths of the undeca­fluoro­diarsenate ([As_2_F_11_]^−^) anion are consistent with values reported in the literature (Minkwitz & Neikes, 1999 ▸).

Within its packing, the benzo­acyl­ium cation is surrounded by six [As_2_F_11_]^−^ anions [Fig. 5 ▸(*b*)] and forms C⋯F contacts, as well as a T-shaped π-inter­action (Table 5 ▸). The six C⋯F contacts formed by the acyl­ium moiety are in the range 2.803 (4)–3.151 (4) Å. Except for one C⋯F contact (C6⋯F3) of 2.997 (5) Å, the inter­actions with the arene ring are weaker considering the F⋯H—C distances of 3.284 (5)–3.451 (5) Å. It is noticeable that the contacts of C2—H1 strongly differ from those of other aromatic contacts, because its contact to the anion has a distance of 3.769 (5) Å. The benzo­acyl­ium cation shows rare T-shaped π-stacking in the crystal structure [π–σ(CO) inter­actions]. The closest contacts of the benzo­acyl­ium cations with itself are 3.394 (ring-plane⋯O1) and 3.428 Å (centroid⋯O1), and can be regarded as medium strong (Janiac, 2000 ▸). The acyl­ium moiety is nearly perpendicular to the centre of neighbouring ring systems [Fig. 5 ▸(*b*)], with deviating angles ranging from 81.82 (O1⋯centroid⋯C5) to 97.86° (O1⋯centroid⋯C3).

### Quantum chemical calculations

The quantum chemical calculations were performed at the aug-cc-pVTZ-level of theory at 298 K with the *GAUSSIAN16* program package (Frisch *et al.*, 2016 ▸).

The structures were opimized using DFT methods for the calculation of vibration frequencies. For futher energetic calculations, such as the mapped electrotatic potential, MP2 methods were applied for more accurate energy values.

**Figure 6:**
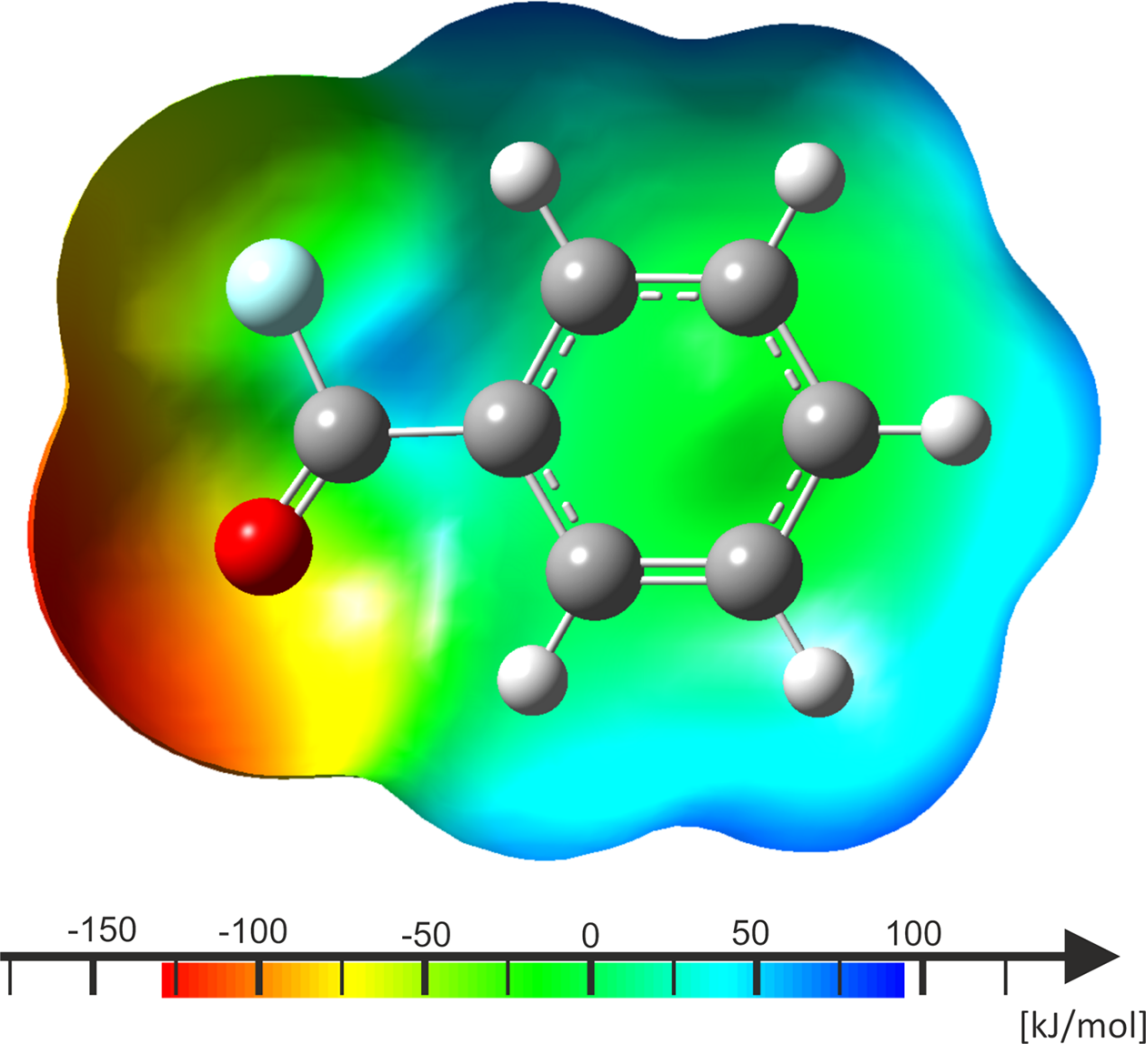
Calculated mapped electrostatic potential onto an electron-density isosurface value of 0.0004 Bohr^−3^, with the colour scale ranging from −127.074 (red) to 87.692 kJ mol^−1^ (blue) of PhCOF.

As depicted in Table 2 ▸, the deviations between the calculated and observed bond lengths are in good agreement. Since the inter­actions within the crystal structure appear to be only weak, no further modelling of contacts was necessary for the calculations. The electron-withdrawing shift towards the substituent can be seen in the mapped electostatic potential (Fig. 6 ▸). The electron-poor carbonyl C atom inhibits an electron hole (blue), as it is attached to the highly electronegative F and O atoms.

**Figure 7:**
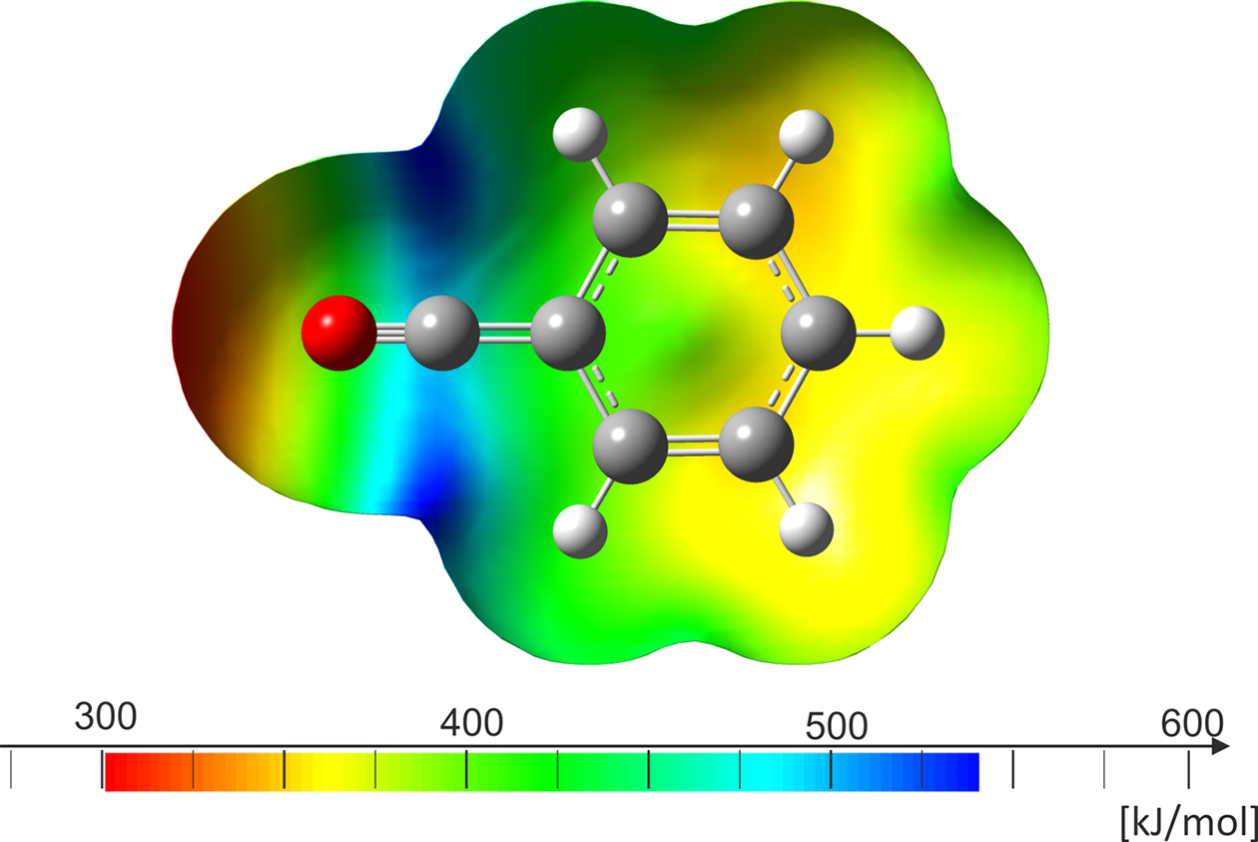
Calculated mapped electrostatic potential onto an electron-density isosurface value of 0.0004 Bohr^−3^, with the colour scale ranging from 301.933 (red) to 538.228 kJ mol^−1^ (blue) of [PhCO][As_2_F_11_].

The calculations for PhCO^+^ are also in accordance with the observed bond lengths, as illustrated in Table 3 ▸, so that values are close to the 3σ rule. The com­parable slightly higher deviation can be rationalized by the influence of stronger inter­actions. As visualized by the mapped electrostatic potential (Fig. 7 ▸), a π-hole (blue) is localized at atom C7.

Comparing the calculations, a similar mapped electrostatic potential has already been calculated for fumaryl fluoride mono­acyl­ium, which posesses both functional groups, *i.e.* acyl­ium and an acyl fluoride moiety (Bayer *et al.*, 2022*a* ▸).

### Vibrational spectroscopy

Experimental vibrational frequencies for benzoyl fluoride and the benzo­acyl­ium cation were assigned according to Tables 6 ▸, 7 ▸ and 8 ▸, in accordance with quantum chemical cal­cul­ations at the B3LYP/aug-cc-pVTZ level of theory, and com­pared to the starting material, benzoic acid (Fig. 1 ▸).

*C*_1_ symmetry was determined for benzoyl fluoride and the benzo­acyl­ium cation, with 36 and 33 fundamental vibrational modes (A), respectively. All observed vibrational frequencies were assigned with the aid of quantum chemical calculations, as listed in Tables 5 ▸ and 6 ▸.

The successful synthesis of the acyl­ium ion is indicated by the stretching vibration of the carbonyl group. The ν(C=O) is assigned to the Raman line at 1634 cm^−1^ in the vibrational spectrum of the starting material and is no longer observed in the vibrational spectrum of **2**. The C≡O stretching vibration of the acyl cation is detected in the Raman spectrum at 2232 cm^−1^ for **2** and in the IR spectrum at 2233 cm^−1^ for **2**. The successful fluoride abstraction was also observed by the absence of the C—F stretching vibration and the COF bending vibrations of the neutral com­pound in the vibrational spectra of **2**. These are detected in the Raman spectrum of the starting material at 1246 and 617 cm^−1^, respectively, but are no longer observed in the vibrational spectra of **2**. The anti­symmetric C—C—OH bending vibration present in the Raman spectrum of benzoic acid at 1324 cm^−1^ was also not detected in the Raman spectra of fluoride **1** and acyl­ium salt **2**. The Raman lines of the C_Ph_—C vibrations were detected blue-shifted from 1150 (benzoic acid) to 1216 (**1**) and 1207 cm^−1^ (**2**). The benzene ring breathing modes are detected in the Raman spectra of **1** and **2** at 1002 and 997 cm^−1^, respectively, and remain unchanged after the transformation of benzoic acid to benzoyl fluoride and fluoride abstraction (5 cm^−1^ blue-shifted). The same trend was observed for ν(C=C), which are not affected by the conversion of benzoic acid to **1** (1602 cm^−1^) and **2** (1583 cm^−1^). The C—H stretching vibrations of the arene ring are observed at 3084, 3075 and 3061 cm^−1^ in **1**, and at 3167, 3108 and 3088 cm^−1^ in **2**, and are red-shifted in com­parison with benzoic acid.

The vibrational frequencies of the [As_2_F_11_]^−^ anions are in accordance with values reported in the literature (Minkwitz & Neikes, 1999 ▸) and are listed in Table 6 ▸.

## Conclusion

Herein we report the first crystal structures of the smallest benzylic acyl fluoride and the acyl cation, as well as their vibrational characterization. The strong carbon bond towards the C—COF or C—CO^+^ moiety, respectively, can be rationalized by the strong strengthening effects of π–π hyperconjugation of the arene subsituent analog to the toluene acyl­ium ion. The strengthening effect is also visable in the blue shift of the Raman lines and is therefore consistent with the calculated values and obtained crystallographic data. Although the com­pounds are stable up to room tem­per­a­ture, the acyl fluoride shows a high volatility even at low tem­per­a­tures.

The challenging crystallization of low-melting volatile com­pounds such as acyl fluorides can succeed starting from saturated solutions with volatile solvents under a cool nitro­gen stream by recrystallization, such as was observed for benzoyl fluoride.

Analogous to the reported benzoic acid derivatives, terephthalic acid and isophthalic acid were reacted, but the products could not be crystallized due to a change of solubility. A change of the solvent thionyl fluoride to 1,1,1,2-tetra­fluoro­ethane (R-134a) or mixtures might lead to successful isolation.

A stabilizing *para*-substituent effect appears not to be necessary when performing the abstraction with anti­mony penta­fluoride. In contrast to the experiments of Davlieva *et al.* (2005 ▸), the acyl cation was obtained instead of the SbCl_5_ adduct. Therefore, it can be deduced that the abstraction of halogenide with anti­mony chloride containing Lewis acids only succeeds for stabilized aromatics, whereas the abstraction with anti­mony penta­fluoride can access acyl cations of less stabilized aromatics.

## Supplementary Material

Crystal structure: contains datablock(s) xk047, xl013, global. DOI: 10.1107/S2053229625000476/ov3178sup1.cif

Structure factors: contains datablock(s) xk047. DOI: 10.1107/S2053229625000476/ov3178xk047sup2.hkl

Structure factors: contains datablock(s) xl013. DOI: 10.1107/S2053229625000476/ov3178xl013sup3.hkl

CCDC references: 2417958, 2417957

## Figures and Tables

**Table 1:** Experimental details For both structures: monoclinic, *P*2_1_/*n*. Experiments were carried out with Mo *K*α radiation using a Rigaku Xcalibur Sapphire3 diffractometer. Absorption was corrected for by multi-scan methods (*CrysAlis PRO*; Rigaku OD, 2020 ▸).

	**1**	**2**
Crystal data		
Chemical formula	C_7_H_5_FO	C_7_H_5_O^+^·As_2_F_11_^−^
*M*_r_	124.11	463.95
Temperature (K)	114	112
*a*, *b*, *c* (Å)	12.592 (3), 7.2274 (17), 13.473 (3)	10.6376 (9), 9.9099 (7), 13.0019 (9)
β (°)	104.77 (2)	101.806 (8)
*V* (Å^3^)	1185.6 (5)	1341.63 (18)
*Z*	8	4
μ (mm^−1^)	0.11	5.11
Crystal size (mm)	0.50 × 0.49 × 0.11	0.40 × 0.32 × 0.25

Data collection		
*T*_min_, *T*_max_	0.151, 1.000	0.213, 1.000
No. of measured, independent and observed [*I* > 2σ(*I*)] reflections	7905, 2413, 1506	13692, 3328, 2658
*R*_int_	0.064	0.053
(sin θ/λ)_max_ (Å^−1^)	0.625	0.667

Refinement		
*R*[*F*^2^ > 2σ(*F*^2^)], *wR*(*F*^2^), *S*	0.080, 0.249, 1.04	0.036, 0.095, 1.06
No. of reflections	2413	3328
No. of parameters	203	206
H-atom treatment	All H-atom parameters refined	H atoms treated by a mixture of independent and constrained refinement
Δρ_max_, Δρ_min_ (e Å^−3^)	0.38, −0.42	0.82, −0.61

Computer programs: *CrysAlis PRO* (Rigaku OD, 2020 ▸), *SHELXT* (Sheldrick, 2015*a* ▸), *SHELXL2018* (Sheldrick, 2015*b* ▸), *ORTEP-3 for Windows* (Farrugia, 2012 ▸) and *PLATON* (Spek, 2020 ▸).

**Table 2:** Inter­atomic distances (Å) for benzoic acid, benzoyl chloride and the two independent rings in benzoyl fluoride ‘Lit’ is literature, ‘Exp’ is experimental and ‘Calc’ is calculated (B3LYP/aug-cc-pVTZ).

PhCO_2_H	Lit	PhCOCl	Lit	PhCOF 1	Exp	PhCOF 2	Exp	Calc
C=O	1.252	C=O	1.177 (3)	C1=O1	1.222 (4)	C14=O2	1.224 (4)	1.186
C—O	1.300	C—Cl	1.787 (2)	C7—F1	1.296 (5)	C14—F2	1.312 (4)	1.367
C1—C2	1.491	C7—C1	1.471 (3)	C7—C1	1.472 (4)	C7—C1	1.472 (3)	1.474
C2—C3	1.405	C1—C2	1.383 (3)	C1—C2	1.380 (5)	C1—C2	1.391 (4)	1.397
C3—C4	1.446	C2—C3	1.385 (3)	C2—C3	1.389 (4)	C2—C3	1.386 (3)	1.388
C4—C5	1.390	C3—C4	1.374 (4)	C3—C4	1.385 (4)	C3—C4	1.377 (4)	1.391
C5—C6	1.367	C4—C5	1.377 (4)	C4—C5	1.374 (5)	C4—C5	1.384 (5)	1.392
C6—C7	1.431	C5—C6	1.379 (3)	C5—C6	1.395 (4)	C5—C6	1.387 (4)	1.385
C2—C7	1.389	C6—C1	1.390 (3)	C6—C1	1.394 (3)	C6—C1	1.383 (4)	1.398

**Table 3:** Contacts (Å) of the benzoacyl cation in the structure of PhCOF (**1**)

Contact	Distance
F1⋯C14^iii^	3.092 (4)
O1⋯(H3)^ii^C4^ii^	3.295 (3)
C2⋯C7^i^	3.328 (5)
C3(H2)⋯O2^ii^	3.398 (5)
C11(H8)⋯F2^ii^	3.461 (3)
C9(H6)⋯O2^iii^	3.520 (4)
C4⋯(H9^iii^)C12^iii^	3.659 (5)
C3⋯(H10^iii^)C13^iii^	3.775 (5)
C1⋯(H10^iii^)C13^iii^	3.779 (4)
C2⋯(H10^iii^)C13^iii^	3.823 (4)

Symmetry codes: (i) −*x* + 1, −*y* + 1, −*z* + 1; (ii) *x* − 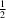, −*y* + 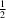, *z* − 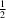; (iii) −*x* + 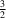, *y* − 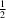, −*z* + 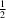.

**Table 4:** Inter­atomic distances (Å) for the benzoacyl cation and the CH_3_CO^+^ cation ‘Exp’ is experimental, ‘Calc’ is calculated (B3LYP/aug-cc-pVTZ) and ‘Lit’ is literature.

PhCO^+^	Exp	Calc	TolCO^+^	Lit	CH_3_CO^+^	Lit
C≡O	1.109 (5)	1.126	C≡O	1.116 (2)	C≡O	1.116
C1—C7	1.403 (5)	1.378	C1—C7	1.391 (2)	C1—C2	1.378 (2)
C1—C2	1.404 (5)	1.417	C1—C2	1.405 (2)		
C2—C3	1.374 (5)	1.377	C2—C3	1.376 (2)		
C3—C4	1.380 (6)	1.397	C3—C4	1.402 (2)		
C4—C5	1.390 (5)	1.397	C4—C5	1.397 (2)		
C5—C6	1.380 (5)	1.377	C5—C6	1.380 (2)		

**Table 5:** Contacts (Å) of the benzoacyl cation in the structure of [PhCO][As_2_F_11_]

Contact	Distance
C7⋯F3	2.803 (4)
C7⋯F11^i^	2.873 (4)
O1⋯F8^ii^	2.893 (3)
C7⋯F9^iii^	2.900 (5)
C7⋯F4^ii^	2.986 (4)
O1⋯F3	2.988 (4)
C6⋯F3	2.997 (5)
C7⋯F7^i^	3.102 (4)
C1⋯F3	3.145 (4)
C4⋯F1^ii^	3.151 (4)
C6(H5)⋯F7^i^	3.284 (5)
C5(H4)⋯F5^iii^	3.417 (5)
C4(H3)⋯F1^iii^	3.451 (5)
O1⋯plane(ring)/O1⋯centroid(C1–C6)	3.394/3.428

Symmetry codes: (i) −*x*, −*y*, −*z*; (ii) −*x* + 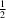, *y* + 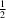, −*z* + 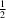; (iii) *x* + 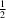, −*y* + 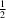, *z* + 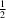.

**Table 6:** Measured and calculated vibration frequencies (cm^−1^) for PhCO_2_H

Raman	Calc^*a*,*b*^ (Raman/IR)^*c*^	Assignment
	3627 (138/94)	ν(O—H)
3073 (42)	3104 (121/2)	ν(C—H)
3063 (28)	3098 (102/4)	ν(C—H)
3039 (5)	3084 (135/12)	ν(C—H)
3009 (6)	3075 (98/10)	ν(C—H)
2982 (4)	3063 (52/0)	ν(C—H)
	1721 (89/367)	ν(C=O)
1634 (18)	1588 (75/18)	ν(C=C)
1602 (32)	1569 (6/5)	ν(C=C)
	1478 (1/1)	δ(C=C)
1443 (4)	1437 (2/15)	δ(C=C)
1324 (7)	1320 (12/115)	δ(C—COH)
	1310 (1/4)	δ(C=C)
1290 (14)	1295 (1/2)	ν(C=C)
1180 (7)	1170 (12/60)	δ(C—H)
1170 (4)	1150 (22/160)	δ(C—H) + ν(C—C)
1158 (4)	1145 (6/1)	δ(C—H)
1133 (6)	1078 (1/41)	δ(C—H)
	1055 (0/119)	δ(C=C)
1028 (14)	1014 (11/19)	δ(C=C)
	992 (0/0)	δ(C—H)
1002 (100)	989 (45/0)	Ring breathing
991 (3)	978 (0/0)	τ(C—H)
	940 (0/1)	τ(C—H)
	845 (0/0)	τ(C—H)
812 (5)	801 (1/0)	δ(C—H)
	750 (18/8)	δ(C—C=C)
	708 (0/123)	τ(C—H)
	685 (0/8)	ω(C—C=C)
618 (14)	618 (1/48)	δ(C—C=C)
	612 (5/0)	δ(C—C=C)
	575 (2/61)	τ(O—H)
	480 (1/6)	δ(C—COH)
421 (10)	423 (0/9)	δ(C—C=C)
	401 (0/0)	ω(C—C=C)
	371 (4/5)	δ(C—C=C)
195 (21)	210 (0/2)	δ(C—CO_2_H)
	153 (2/1)	δ(CO_2_H)
	60 (0/1)	ω(CO_2_H)

Notes: (*a*) calculated at the B3LYP/aug-cc-pVTZ level; (*b*) scaling factor 0.967; (*c*) IR intensities in kJ mol^−1^ and Raman intensities in Å^4^/AMU or % at observed frequencies.

**Table 7:** Measured and calculated vibration frequencies (cm^−1^) for PhCOF

Raman	Calc^*a*,*b*^ (Raman/IR)^*c*^	Assignment
	3107 (120/2)	ν(C—H)
	3098 (121/4)	ν(C—H)
3084 (21)	3086 (115/9)	ν(C—H)
3075 (27)	3078 (95/8)	ν(C—H)
3061 (9)	3066 (51/0)	ν(C—H)
1809 (48)		
1795 (31)	1797 (132/404)	ν(C=O)
1758 (36)		
1602 (100)	1587 (75/24)	ν(C=C)
1589 (11)	1569 (5/2)	ν(C=C)
1494 (3)	1477 (0/1)	ν(C=C)
1457 (3)	1437 (1/14)	ν(C=C)
1323 (3)	1312 (1/4)	δ(C—H)
1268 (13)	1296 (0/2)	ν(C=C)
1246 (13)	1216 (33/217)	ν(C—COF)
1178 (8)	1161 (5/27)	δ(C—H)
1167 (18)	1147 (5/1)	δ(C—H)
	1073 (1/2)	δ(C=C)
1018 (10)	1019 (6/16)	δ(C=C)
1011 (7)	995 (0/0)	ν(C—F)
	990 (23/165)	δ(C=C)
1002 (98)	988 (28/22)	Ring breathing
	978 (0/0)	δ(C—H)
	941 (0/1)	δ(C—H)
855 (2)	844 (0/0)	δ(C—H)
787 (9)	793 (1/3)	δ(C—H)
771 (28)	749 (17/15)	δ(C—C=C)
	696 (0/96)	δ(C—H)
	678 (0/1)	δ(C—C=C)
	632 (0/17)	δ(C—C=C)
617 (21)	611 (5/1)	δ(C—COF)
492 (3)	477 (2/1)	δ(C—C=C)
	427 (0/0)	ω(C—C=C)
	401 (0/0)	τ(C—C)
382 (15)	366 (4/3)	δ(C—C=C)
217 (4)	205 (0/1)	δ(C—COF)
187 (24)		
173 (21)	153 (2/0)	δ(C=O)
	64 (1/0)	τ(COF)

Notes: (*a*) calculated at the B3LYP/aug-cc-pVTZ level; (*b*) scaling factor 0.967; (*c*) IR intensities in kJ mol^−1^ and Raman intensities in Å^4^/AMU or % at observed frequencies.

**Table 8:** Measured vibrations for [PhCO][As_2_F_11_] and calculated vibration frequencies (cm^−1^) for [PhCO]^+^

Raman	Calc^a,b^	(Raman/IR)^*c*^	Assignment
3167 (5)	3167 (*s*)	3109 (302/1)	ν(C—H)
3144 (5)		3107 (6/12)	ν(C—H)
3137 (5)		3098 (37/6)	ν(C—H)
3108 (14)	3107 (*s*)	3096 (91/0)	ν(C—H)
3088 (23)	3084 (*s*)	3087 (42/0)	ν(C—H)
2253 (8)			
2232 (46)	2233 (*s*)	2211 (144/930)	ν(C≡O)
2223 (63)			
1583 (100)	1601 (*s*)	1564 (51/152)	ν(C=C)
		1536 (1/0)	ν(C=C)
1451 (5)	1450 (*s*)	1455 (2/2)	ν(C=C)
		1428 (1/43)	ν(C=C)
1328 (4)	1321 (*s*)	1330 (1/16)	ν(C=C)
		1292 (0/1)	ν(C=C)
1182 (13)	1192 (*s*)	1207 (2/54)	ν(C—CO)
1177 (10)	1178 (*s*)	1166 (4/3)	δ(C=C)
1158 (15)		1164 (3/65)	ν(C—CO)
1104 (4)		1085 (1/1)	ν(C=C)
1021 (16)	1030 (*s*)	1020 (0/0)	ν(C=C)
		1002 (16/0)	δ(C=C)
		984 (0/0)	τ(C—H)
997 (74)	999 (*s*)	974 (36/11)	Ring breathing
		951 (0/1)	τ(C—H)
		823 (0/0)	τ(C—H)
763 (15)			
751 (13)		755 (0/42)	δ(C—H)
740 (17)		748 (21/1)	δ(C—CO)
725 (7)	696 (*vs*)	646 (0/32)	
639 (12)		636 (2/4)	δ(C—CO)
609 (10)		584 (3/3)	δ(C—CO)
		583 (0/27)	δ(CO)
452 (43)		442 (15/4)	δ(C—C=C)
		378 (0/0)	ω(C—C=C)
370 (8)		370 (0/0)	δ(C—H)
311 (12)			
172 (12)			
160 (16)			
152 (13)		147 (1/2)	δ(C≡O)
		125 (1/0)	δ(C—CO)
			
As_2_F_11_			
740 (17)			ν(As—F)
685 (60)	685 (*s*)		ν(As—F)
586 (9)			δ(As—F)
393 (11)			δ(As—F)

Notes: (*a*) calculated at the B3LYP/aug-cc-pVTZ level; (*b*) scaling factor 0.967; (*c*) IR intensities in kJ mol^−1^ and Raman intensities in Å^4^/AMU or % at observed frequencies.
